# Supplementary material for: Photon pair generation in hydrogenated amorphous silicon microring resonators
Source: Sci Rep. 2016 Dec 20;6:38908. doi: 10.1038/srep38908 (PMC5171649; doi:10.1038/srep38908)
Supplement: Supplementary Information [file srep38908-s1.pdf]

Photon pair generation in hydrogenated amorphous silicon microring resonators

E. Hemsley[1], D. Bonneau[1], J. Pelc[2], R. Beausoleil[2], J. L. O'Brien[1], M. G. Thompson[1]

1. Centre for Quantum Photonics, H. H. Wills Physics Laboratory, University of Bristol, Bristol, UK
2. Hewlett-Packard Laboratories, 1501 Page Mill Rd., Palo Alto, CA, USA

1 Measurement Stability

Figure 1 shows an example of the normalised output power of the pump during the measurement of $Q(P)$ and the measurement of the SFWM rate. In the pump-probe experiment, whilst the high power pump was coupled into the micro-ring, scanning the probe wavelength introduced additional noise to the system. The coupled power in the ring fluctuated during the measurement on average 4.3%, although for highest powers the value was closer to 10%. During the measurement of the SFWM rate the coupled power was more stable, with only 0.12% deviation from the average. The difference in stability between the two experiments is the likely origin of the discrepancy between the extracted values for $\sigma\tau\beta_{SPA}$.

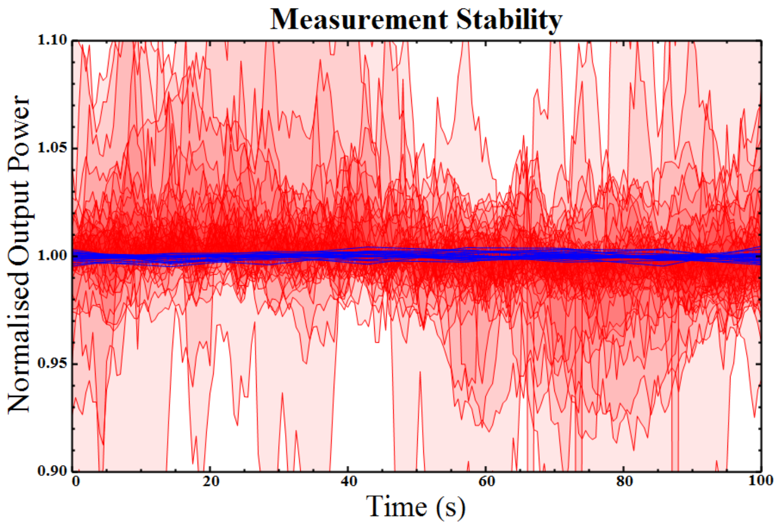

Figure 1: Stability during measurement of a-Si:H ring A. The red lines show the normalised output power of the pump during Q factor measurement with the pump-probe set-up, and the blue lines during measurement of the photon pair generation rate.
